# Functional brain alterations associated with acupuncture for chronic pain: a scoping review of fMRI studies

**DOI:** 10.3389/fnins.2026.1819418

**Published:** 2026-05-21

**Authors:** Yuxin Wu, Leyang Song, Yunyang Zhang, Suwei Liu, Haoyang Yu, Feifei Hao, Yuxia Ma, Na Zhang

**Affiliations:** 1 School of Acupuncture-Moxibustion and Tuina, Shandong University of Traditional Chinese Medicine, Jinan, China; 2 School of Traditional Chinese Medicine, Shandong University of Traditional Chinese Medicine, Jinan, China; 3 Key Laboratory of Traditional Chinese Medicine Classical Theory, Ministry of Education, Shandong University of Traditional Chinese Medicine, Jinan, China

**Keywords:** acupuncture, chronic pain, default mode network, functional magnetic resonance imaging, sensorimotor network

## Abstract

**Background:**

Chronic pain (CP) is a public health challenge recognized as involving large-scale functional brain dysregulation. Acupuncture is widely used as a non-pharmacological intervention for CP, yet its central mechanisms remain incompletely understood. fMRI provides an approach for investigating acupuncture-related brain alterations in CP.

**Methods:**

Eight databases were searched from inception to March 27, 2025 for fMRI studies investigating acupuncture’s central effects in CP. Eligible studies included randomized controlled trials and observational studies involving migraine, knee osteoarthritis, fibromyalgia, sciatica, chronic shoulder pain, chronic neck pain, cervical spondylosis, chronic low back pain, and lumbar disk herniation. Data on characteristics, acupuncture protocols, neuroimaging findings, and outcomes were extracted and narratively synthesized. Reporting quality of acupuncture interventions was assessed using STRICTA, risk of bias of randomized controlled trials using RoB 2, and methodological quality of observational studies using the Newcastle–Ottawa Scale.

**Results:**

A total of 64 studies were included. CP was characterized by widespread functional brain abnormalities, mainly involving the default mode network, sensorimotor network, and pain- and emotion-related regions such as the anterior cingulate cortex, precuneus, insula, and thalamus. Across longitudinal and controlled analyses, acupuncture-related brain changes were most consistently reflected in altered functional connectivity, local neural synchrony, and regional spontaneous activity. Functional connectivity findings suggested a potentially ACC-centered circuit pattern, whereas regional homogeneity findings revealed bidirectional modulation across multiple brain regions. Comparative evidence further indicated that VA, SA, and EEA may engage partially overlapping but distinct neural processes. Reporting of core acupuncture protocol components was generally adequate, whereas methodological quality remained heterogeneous.

**Conclusion:**

Current fMRI evidence suggests that CP involves large-scale network-level functional imbalance and that acupuncture may be associated with modulation of key abnormal nodes and circuits related to pain perception, sensory processing, and emotional regulation. The available evidence supports a cautious interpretation that acupuncture-related brain effects may predominantly reflect a state-dependent recalibration of dysregulated brain networks. Future studies should prioritize large-sample, multicenter, longitudinal, and multimodal designs, together with rigorous control settings and more rigorous, externally validated machine learning-based prediction studies, to better distinguish differential central effects across intervention conditions and advance mechanism-informed personalized acupuncture in CP management.

## 1 Introduction

Chronic pain (CP) is a major global health burden, defined by the World Health Organization as persistent or recurrent pain lasting beyond 3 months (Barke et al., 2020). Epidemiological data suggest that roughly one-third of adults suffer from CP symptoms, with prevalence rates varying by sociodemographic factors such as age, gender, and educational attainment (Rometsch et al., 2025). Approximately 40% of CP patients present with comorbid mental disorders such as anxiety and depression. This condition not only severely impairs patients’ psychological well-being but may also lead to physical functional limitations or even disability, significantly compromising their daily living and work capacity (Wong et al., 2020; Aaron et al., 2025; Sakuma et al., 2025). Current pharmacological management of CP primarily relies on opioids, which are limited by high rates of adverse effects and suboptimal symptom control. Consequently, clinical practice is actively exploring non-pharmacological interventions to develop more systematic and safer pain management strategies (Wuyts et al., 2025; Wang X. et al., 2025; Wang Y. et al., 2025; Ihns et al., 2024).

As a fundamental component of traditional medicine, acupuncture is widely applied in the treatment of pain (Lin et al., 2025) and has demonstrated favorable safety profiles and medium-to-long-term efficacy (Zhu et al., 2025). Studies have shown that acupuncture can effectively alleviate various pain conditions, including knee osteoarthritis (KOA) (Cattarinussi et al., 2024), chronic neck pain (CNP) (Zhao et al., 2024), headache (HA) (Wang et al., 2011), chronic low back pain (CLBP) (Zhou et al., 2024), and sciatica (SCT) (Tu et al., 2024). Furthermore, it alleviates emotional disturbances accompanying pain, including anxiety and depression (Lou and Bu, 2025), highlighting its unique value in a holistic approach addressing both physical and mental aspects.

Advancements in neuroimaging have provided scientific insights into the mechanisms underlying acupuncture analgesia (Peihong et al., 2021). Research indicates that acupuncture can modulate key brain regions, including the Prefrontal Cortex (PFC) and anterior cingulate cortex (ACC), and enhance neural circuit connectivity, such as between the PFC and the Pregenual Anterior Cingulate Cortex / Periaqueductal Gray (PAG), thereby exerting integrated regulatory effects on the medial pain system and the limbic system (Qu et al., 2024). This multi-level neuromodulatory profile may contribute to pain-related symptom modulation and may also be relevant to pain-associated cognitive and emotional dysfunctions, providing a useful perspective for understanding the potential regulatory role of acupuncture in CP management. As a noninvasive neuroimaging tool, fMRI is essential for investigating the central nervous system mechanisms of acupuncture (Li et al., 2022). Current research predominantly utilizes resting-state fMRI (rs-fMRI), while task-based fMRI (t-fMRI) being relatively less common.

In the field of acupuncture fMRI research, current investigations primarily focus on pain mechanisms. However, the existing literature in this domain is generally constrained by methodological limitations, including insufficient standardization of experimental designs and a lack of quality control systems (Zhang et al., 2020), which substantially undermine the reliability and replicability of research findings. This article aims to promote the evolution of acupuncture fMRI research toward standardized experimental design and transparent data analysis, to clarify the potential central pathogenesis of CP in the brain and the potential neural mechanisms by which acupuncture modulates brain function and structure in CP patients, and ultimately to achieve effective integration of neuroimaging evidence with clinical practice.

## 2 Materials and methods

### 2.1 Literature retrieval

A systematic search was conducted across eight databases: China National Knowledge Infrastructure (CNKI), VIP Chinese Journal Full-text Database, Wanfang Data Knowledge Service Platform, Chinese Biomedical Literature Database, PubMed, Embase, Cochrane Library, and Web of Science. The search encompassed fMRI studies related to acupuncture treatment for CP, from the inception of each database until March 27, 2025.

The target diseases for this review included migraine (MIG), KOA, fibromyalgia (FM), SCT, chronic shoulder pain (CSP), CNP, cervical spondylosis (CS), CLBP and lumbar disk herniation (LDH). A combined search strategy utilizing subject headings and free-text terms was employed.

### 2.2 Inclusion and exclusion standards

#### 2.2.1 Inclusion standards

(1) Study types: Randomized controlled trials (RCTs), observational studies (including prospective or retrospective cohort studies, or cross-sectional studies);

(2) Participants: Patients diagnosed with CP conditions including MIG, KOA, FM, SCT, CSP, CNP, CS, CLBP, or LDH; healthy controls (HC) were also included.

(3) Study groups:

For RCTs: The intervention group received traditional acupuncture treatment (with no restrictions on acupuncture type, manipulation techniques, treatment frequency, or presence of psychological intervention). The control group consisted of CP patients receiving sham acupuncture, waitlist control, conventional medication, or other forms of control.

For interpretive purposes, comparator conditions were considered in three categories: sham-related controls, inactive or usual-care controls, and expectancy- or context-enhanced intervention conditions. This stratification was used to improve the interpretability of between-group findings and to reduce inappropriate conflation of acupuncture-specific, nonspecific, and context-related central effects.

For observational studies: Cross-sectional comparisons between patient groups and HC groups; or within-subject comparisons (pre-vs. post-intervention) in patient groups only.

(4) At least one fMRI scan was acquired before/after the acupuncture intervention or during the acupuncture intervention.

#### 2.2.2 Exclusion criteria

(1) The target condition was not a CP disorder;

(2) The full text was unavailable or unreadable;

(3) The intervention did not satisfy the eligibility criteria;

(4) The neuroimaging outcome measures did not meet the inclusion criteria;

(5) Publications were restricted to those in the English or Chinese languages.

### 2.3 Literature selection and data extraction

#### 2.3.1 Literature screening process

EndNote 21 was used to manage the literature screening process. Following duplicate removal, two reviewers independently examined titles and abstracts to eliminate irrelevant records. Full texts of potentially eligible articles were then retrieved and assessed against inclusion criteria. Screening results were compared between reviewers, with final inclusion decisions reached through consensus. In cases of disagreement, a third senior researcher was consulted to resolve disputes, ensuring objectivity and methodological rigor throughout the selection process.

#### 2.3.2 Data abstraction

Data from the finally included studies was extracted using Excel. The extracted information included:

(1) Basic study characteristics: title, first author, corresponding author, publication year, country, etc.;

(2) Experimental Design Parameters: study design type, disease duration of included patients, and sample size of enrolled patients;

(3) Intervention Protocol Details: type of acupuncture intervention, duration of treatment course, and acupuncture grouping methodology;

(4) Neuroimaging Parameters: data analysis techniques, and alterations in brain regions before and after the intervention;

(5) Efficacy Evaluation Framework: clinical outcome assessment metrics, and related measures.

#### 2.3.3 Reporting and methodological quality assessment

Given that this scoping review included both randomized controlled trials and observational studies, the completeness of reporting and methodological quality were assessed using tools appropriate to the study design. The quality of acupuncture intervention reporting was evaluated using the STRICTA guidelines; the risk of bias in randomized controlled trials was appraised using the Cochrane RoB 2 tool; and the methodological quality of observational studies was assessed using the NOS. All assessments were performed independently by two reviewers, and any discrepancies were resolved through discussion or adjudication by a third reviewer. These assessments were employed to characterize the quality features of the included evidence rather than to serve as criteria for study exclusion.

## 3 Results

**Figure 1:**
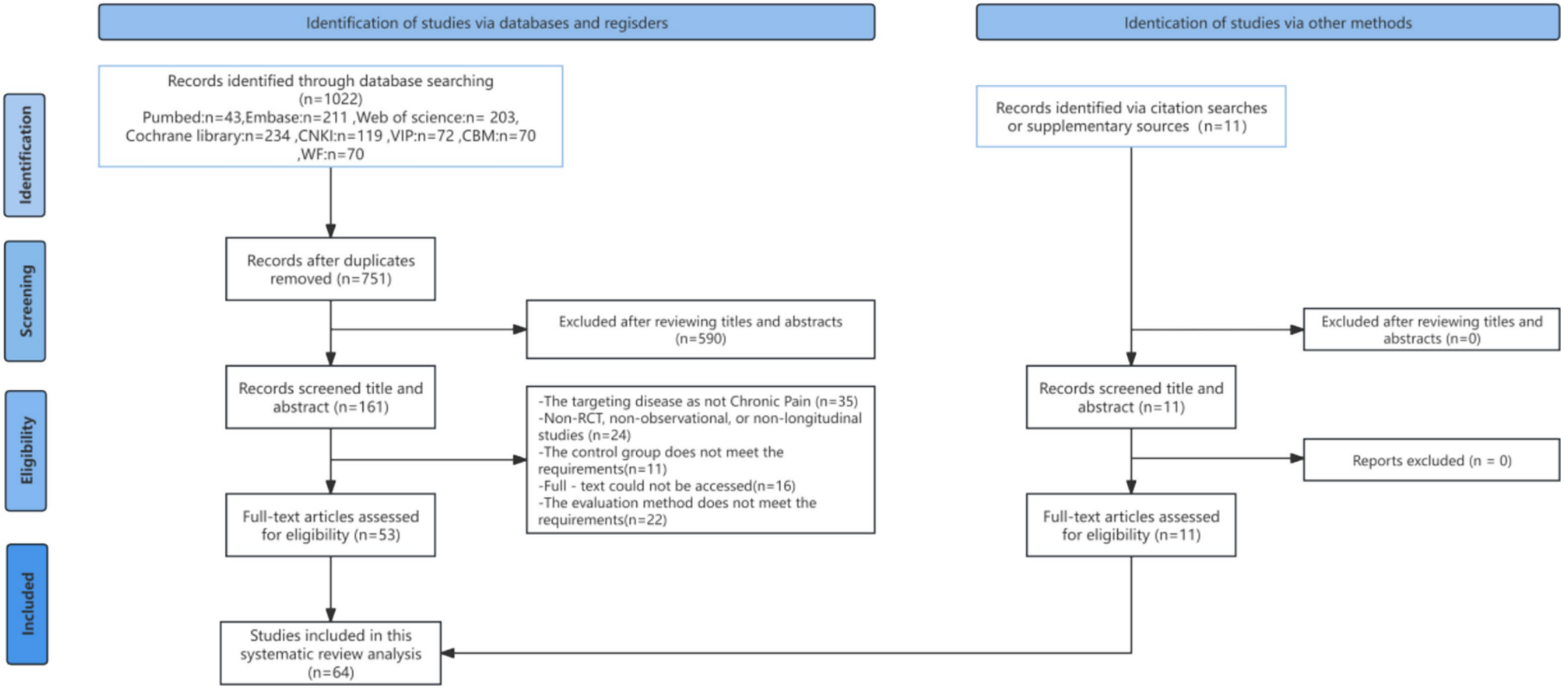
Flowchart of study selection for the scoping review, including record numbers at each stage and reasons for exclusion.

The initial search across eight databases, including CNKI, PubMed, and EMBASE, yielded 1,022 records. After removing 271 duplicates, the titles and abstracts of 751 articles were screened. Of these, 161 studies underwent full-text assessment for eligibility. During this stage, 35 studies were excluded due to non-CP conditions, 24 were excluded for not meeting the study type criteria, 11 were excluded due to ineligible control groups, 16 were excluded because the full text was unavailable, and 22 were excluded due to outcome measures not meeting the inclusion criteria. An additional 11 records were identified through manual searches of reference lists and other sources. Consequently, this review yielded a final inclusion of 64 relevant studies (the literature selection process is detailed in Figure 1).

### 3.1 Publication characteristics

**Figure 2:**
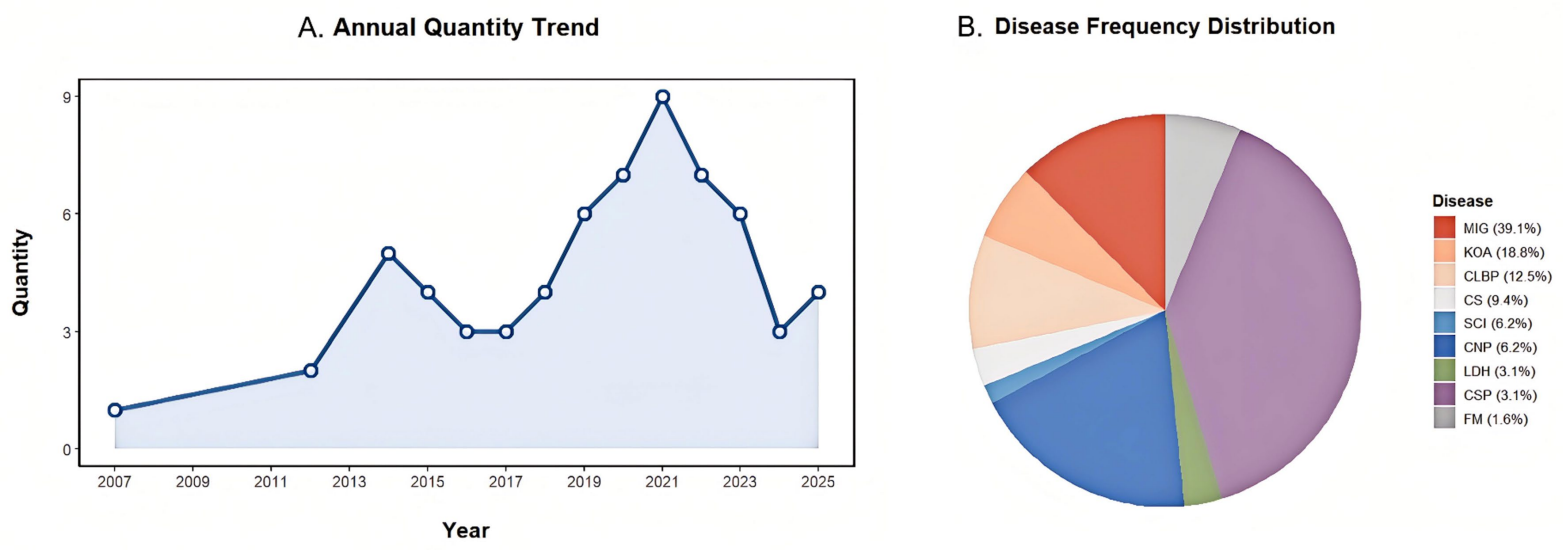
**(A)** Annual quantity from 2007 to 2025. **(B)** Pie chart of disease frequency proportions.

The annual number of published fMRI studies on acupuncture for CP demonstrated distinct phased trends: a period of slow growth from 2007 to 2014 (1 to 5 articles annually), followed by a phase of rapid increase from 2014 to 2021 (Figure 2A). Of the 64 included studies, 52 originated from China, 10 from the United States, and 2 from South Korea. The collection comprised 33 observational studies and 31 randomized controlled trials. Categorized by disease type, the publications consisted of 25 studies on MIG, 12 on KOA, 1 on FM, 4 on SCT, 2 on CSP, 4 on CNP, 6 on CS, 8 on CLBP, and 2 on LDH (Figure 2B). Table 1 presents the fundamental details of the included literature.

**Table 1:** Basic information of literature.

Literature information		Sample information					Acupuncture information			fMRI information	
Author (year)	Country	Study design	Disease	Duration	Sample size (EG/CG)	Healthy sample Size	Courses	Intervention type	Grouping	Imaging condition	Analytical approaches
Shen et al. (2021b)	CHN	Obs.	CS-C	≥6 months	16(16/0)	16	1×	MA	VA vs. HC	rs-fMRI	FC
Cai et al. (2020)	CHN	Obs.	CS-VA	None	12(12/0)	None	1×	MA	VA	rs-fMRI	FC
Gao et al. (2025b)	CHN	RCT	CS	>3 months	40(20/20)	40	2 wk	MA	VA vs. SA vs. HC	rs-fMRI	FC fALFF
Chen W. C. et al. (2015)	CHN	RCT	CS-C	None	49(25/24)	19	4 wk	EA	Single VA vs. Multi-VA vs. HC	rs-fMRI	ReHo
Hou et al. (2014)	CHN	RCT	CS	None	49(25/24)	19	4 wk	EA	Single VA vs. Multi-VA vs. HC	rs-fMRI	ReHo
Shen et al. (2021a)	CHN	Obs.	CS-C	≥6 months	16(16/0)	16	1×	MA	VA vs. HC	rs-fMRI	FC
Yan et al. (2020)	CHN	RCT	CSP	6 weeks to 1 year	24 (12/12)	None	1×	MA	Single-VA vs. Multi-VA	rs-fMRI	DC
Zhang et al. (2018)	CHN	RCT	CSP	6 weeks to 1 year	24 (12/12)	None	1×	MA	Single-VA vs. Multi-VA	rs-fMRI	ReHo
Xu et al. (2021a)	CHN	RCT	CNP	≥3 months	73(39/34)	26	4 wk	MA	VA vs. RCG vs. HC	rs-fMRI s-MRI	FC VBM
Gao et al. (2025a)	CHN	Obs.	CNP	None	80(80/0)	None	2 wk	MA	High-VA vs. Low-VA	rs-fMRI	FC ALFF
Wang et al. (2024)	CHN	RCT	CNP	>3 months	99(66/33)	None	4 wk	MA	VA vs. SA	rs-fMRI	FC
Xu H. et al. (2022)	CHN	Obs.	CNSP	≥6 months	30(30/0)	30	4 wk	MA	VA vs. HC	rs-fMRI	FC
Zou et al. (2019b)	CHN	Obs.	CLBP	≥3 months	60(60/0)	30	4 wk	EA	KD-VA vs. Non-KD-VA vs. HC	rs-fMRI	ReHo
Jia R. H. et al. (2021)	CHN	Obs.	CLBP	2 months to 1 year	20(20/0)	None	2 wk	EA	VA	rs-fMRI	FC
Yu et al. (2020)	USA	RCT	CLBP	≥6 months	79 (Not Reported)	None	4 wk	MA	EC-VA vs. LC-VA vs. EC-SA vs. LC-SA	rs-fMRI	FC
Li et al. (2014)	CHN	Obs.	CLBP	≥3 months	20(20/0)	10	4 wk	MA	VA vs. HC	rs-fMRI	FC ICA
Lee J. et al. (2019)	KR	RCT	CLBP	None	56(33/23)	None	1×	MA	VA vs. SA	rs-fMRI t-fMRI	FC ICA
Tu et al. (2019)	USA	RCT	CLBP	≥6 months	79 (Not Reported)	None	4 wk	MA	EC-VA vs. LC-VA vs. EC-SA vs. LC-SA	rs-fMRI	FC ICA
Makary et al. (2018)	KR	RCT	CLBP	None	56(33/23)	None	1×	MA	VA vs. SA	rs-fMRI t-fMRI	GLM
Kim et al. (2020)	USA	RCT	CLBP	>6 months	102 (Not Reported)	50	4 wk	MA	VA vs. SA vs. SL-VA vs. RC vs. HC	rs-fMRI s-MRI	VBM DTI
Luo et al. (2022)	CHN	Obs.	MIG	>1 year	20(20/0)	20	1×	EA	VA vs. HC	rs-fMRI t-fMRI	FC GLM
Jia et al. (2021b)	CHN	Obs.	MIG	≥1 year	15(15/0)	None	4 wk	MA	VA	rs-fMRI	ReHo
Jia et al. (2021a)	CHN	Obs.	MIG	≥1 year	15(15/0)	None	4 wk	MA	VA	rs-fMRI	FC
Hou et al. (2025)	CHN	Obs.	MIG	None	50(30/20)	None	4 wk	MA	High-VA vs. Low-VA	rs-fMRI	dFC
Xu et al. (2023)	CHN	Obs.	MIG	≥1 year	20(20/0)	20	4 wk	MA	VA vs. HC	rs-fMRI	DC
Liu et al. (2016)	CHN	Obs.	MIG	>1 year	15(15/0)	13	1×	MA	VA vs. HC-VA	rs-fMRI t-fMRI	FC
Qin et al. (2019)	CHN	RCT	MIG	None	40(20/20)	None	4 wk	MA	VA vs. SA	rs-fMRI	ReHo
Liu S. S. et al. (2022)	CHN	Obs.	MIG	None	34(34/0)	16	6 wk	ME+EA	VA vs. HC	rs-fMRI	FC
Ning et al. (2020)	CHN	Obs.	MIG	>1 year	19(19/0)	18	1×	MA	VA vs. HC	rs-fMRI t-fMRI	ALFF
Xiong and Li (2025)	CHN	Obs.	MIG	>1 year	16(16/0)	16	1×	MA	VA vs. HC-VA	rs-fMRI t-fMRI	DC
Zhang Y. N. et al. (2024)	CHN	Obs.	MIG	≥1 year	28(28/0)	None	1×	MA	VA	rs-fMRI	ReHo VMHC
Quan et al. (2024)	CHN	Obs.	MIG	None	143(143/0)	100	8 wk	EA	VA vs. HC	rs-fMRI	dFNC
Li et al. (2017b)	CHN	RCT	MIG	≥6 months	100 (Not Reported)	46	4 wk	MA	VA vs. SA vs. WG vs. HC	rs-fMRI	ALFF
Tian et al. (2021)	CHN	Obs.	MIG	≥6 months	52 (52/0)	60	4 wk	MA	VA vs. HC	rs-fMRI	FC GLM
Zou et al. (2019a)	CHN	Obs.	MIG	>3 months	35(35/0)	25	12 wk	MA	VA vs. HC	rs-fMRI	FC ICA
Zhang et al. (2016)	CHN	Obs.	MIG	>1 year	12(12/0)	12	4 wk	MA	VA vs. HC	rs-fMRI	FC ICA
Li et al. (2017a	CHN	RCT	MIG	>6 months	100(60/40)	60	4 wk	MA	VA vs. SA vs. WG vs. HC	rs-fMRI	FC ICA GLM
Yang et al. (2020)	CHN	RCT	MIG	≥1 year	110 (55/55)	None	4 wk	MA	High-VA vs. Low-VA vs. SA	s-MRI	VBM
Li et al. (2020)	CHN	RCT	MIG	≥6 months	100(60/40)	46	4 wk	MA	VA vs. SA vs. WG vs. HC	rs-fMRI	fALFF
Liu L. et al. (2022)	CHN	RCT	MIG	≥1 year	64 (32/32)	None	4 wk	MA	VA vs. SA	rs-fMRI	FC
Chen et al. (2022)	CHN	Obs.	MIG	>6 months	40(40/0)	36	6 wk	ME+EA	VA vs. HC	rs-fMRI	dALFF GCA
Liu et al. (2021)	CHN	Obs.	MIG	>6 months	40(40/0)	16	6 wk	ME+EA	VA vs. HC	rs-fMRI	ReHo
Li et al. (2023)	CHN	RCT	MIG	≥1 year	38(12/26)	10	10 D	MA	VA vs. SA vs. WL vs. HC	rs-fMRI	ALFF fALFF ReHo
Li et al. (2015)	CHN	Obs.	MIG	>1 year	12(12/0)	12	4 wk	MA	VA vs. HC	rs-fMRI s-MRI	FC ICA DTI
Zhao et al. (2014)	CHN	RCT	MIG	None	80(40/40)	None	8 wk	MA	VA vs. SA	rs-fMRI	ReHo
Liu et al. (2023)	CHN	Obs.	KOA	None	25(25/0)	None	3 wk	MA	VA	rs-fMRI	FC
Qu et al. (2021)	CHN	Obs.	KOA	None	80(80/0)	80	30 D	MA	VA vs. HC	rs-fMRI	ALFF
Zhou et al. (2022)	CHN	RCT	KOA	None	72(36/36)	None	2 wk	MA	VA vs. SA	rs-fMRI	FC ALFF ReHo
Gao et al. (2021)	CHN	Obs.	KOA	≥4 months	15(15/0)	15	1×	MA	VA vs. HC	rs-fMRI	FC
Chen et al. (2014)	USA	RCT	KOA	None	44 (Not Reported)	None	4 wk	MA	HD-VA vs. LD-VA vs. HD-SA	rs-fMRI s-MRI	FC GLM VBM
Kong et al. (2018)	USA	RCT	KOA	None	74 (Not Reported)	None	8 wk	MA	EEG-VA vs. SG-VA vs. RTG	rs-fMRI	FC
Hashmi et al. (2014)	USA	RCT	KOA	≥3 months	67 (Not Reported)	None	1×	EA	VA vs. SA	rs-fMRI	FC DC
Gollub et al. (2018)	USA	RCT	KOA	None	67 (Not Reported)	None	1×	EA	VA vs. SA	rs-fMRI t-fMRI	GLM ReHo
Chen X. et al. (2015)	USA	RCT	KOA	None	44 (Not Reported)	None	4 wk	MA	HD-VA vs. LD-VA vs. HD-SA	t-fMRI	FC GLM ICA
Zhou et al. (2023)	CHN	RCT	KOA	>3 months	180(36/144)	41	2 wk	MA	VA vs. SA vs. SC vs. PB vs. WL vs. HC	rs-fMRI	FC
Wang et al. (2023)	CHN	RCT	KOA	≥6 months	90(30/60)	None	4 wk	MA	VA vs. SA vs. WG	rs-fMRI t-fMRI	fALFF
Egorova et al. (2015)	USA	RCT	KOA	None	44 (Not Reported)	None	4 wk	MA	HD-VA vs. LD-VA vs. SA	rs-fMRI	FC
Napadow et al. (2012)	USA	Obs.	FM	>1 year	17(17/0)	None	4 wk	MA	VA vs. SA	rs-fMRI	FC GLM ICA
Liu et al. (2017)	CHN	Obs.	LDH	None	10(10/0)	None	1×	MA	VA	rs-fMRI t-fMRI	ALFF
Xiao and Wang (2017)	CHN	RCT	LDH	1 month to 10 years	92(42/50)	None	1×	MA	LTA-VA vs. TA-VA	rs-fMRI	ReHo
Li et al. (2012)	CHN	Obs.	SCT	>3 months	10(10/0)	10	4 wk	EA	VA vs. HC	rs-fMRI	ICA
Li et al. (2007)	CHN	Obs.	SCT	None	12(12/0)	None	1×	EA	AA-VA vs. UA-VA	rs-fMRI t-fMRI	GLM
Wei et al. (2024)	CHN	RCT	SCT	>3 months	60 (30/30)	None	4 wk	MA	VA vs. SA	rs-fMRI	fALFF
Liu et al. (2020)	CHN	Obs.	SCT	≥2 weeks	12(12/0)	15	4 wk	MA	VA vs. HC	rs-fMRI	FC ReHo

Group classification: EG (Experimental Group); CG (Control Group).

### 3.2 Reporting quality and methodological quality assessment

#### 3.2.1 STRICTA guideline assessment

This review employed the STRICTA checklist to evaluate the reporting quality of acupuncture interventions across the 64 included studies. Overall, the completeness of reporting for core acupuncture protocol components was largely adequate. All studies fully reported four key items, including the type of acupuncture administered, mode of needle stimulation, total number of treatment sessions, and treatment frequency and duration per session. Furthermore, the majority of studies clearly described the therapeutic rationale, scope of individualized protocol adjustments, mean number of needles inserted per subject per session, acupoint nomenclature, anticipated therapeutic effects, needle retention time, and treatment setting and context. Nevertheless, reporting deficiencies were evident for several items, most notably needle insertion depth, needle specifications, practitioner background information, justification for the choice of control intervention, and detailed description of the control protocol, with only two studies reporting co-interventions administered alongside acupuncture. Collectively, while the existing studies adequately delineate the principal framework of acupuncture protocols, they lack a substantial number of critical details essential for study replicability and scientific interpretation of findings (see Supplementary Table 1).

#### 3.2.2 Risk of bias assessment for randomized controlled trials

The risk of bias in randomized controlled trials was appraised using the RoB 2 tool. The overall methodological quality of the included RCTs varied considerably. The majority of studies exhibited a low risk of bias arising from the randomization process and from deviations from intended interventions, and the overall control of selective outcome reporting was relatively favorable. However, considerable concerns were noted in the domains of missing outcome data and outcome measurement, which constituted the primary sources of variability in the overall risk of bias judgments across studies. Consequently, the internal validity of the included RCTs is inconsistent, with pronounced heterogeneity in overall risk of bias (see Supplementary Figures S1, S2).

#### 3.2.3 Methodological quality assessment for observational studies

The methodological quality of observational studies was evaluated using the NOS. Marked variability in methodological quality was evident across different observational study designs. Case–control studies and prospective cohort studies were generally of moderate to high quality, with several studies achieving favorable overall scores. In contrast, scores for single-group pre-post observational studies were universally low, primarily constrained by inadequate between-group comparability and weak control for various potential sources of bias. These findings indicate that while observational studies can provide valuable exploratory evidence and longitudinal data, the inferential value differs substantially across study designs (see Supplementary Table 2).

### 3.3 Study sample characteristics

#### 3.3.1 Sample size

Among the 64 included studies, a total of 3,257 patients with chronic pain were recruited, with a mean sample size of 50.89 cases per study (range: 10–180 cases). Among them, 32 studies established a healthy control group, enrolling a total of 944 healthy participants, with a mean of 29.5 subjects per group (range: 10–100 subjects).

#### 3.3.2 Disease duration criteria

Substantial heterogeneity was observed in the disease duration criteria across the 64 included studies. The most frequently specified criterion were “pain duration exceeding 12 months” (14 studies, 21.9%), followed by “exceeding 6 months” (13 studies, 20.3%). Notably, 21 studies (32.8%) did not explicitly define the disease duration criteria.

### 3.4 Acupuncture intervention characteristics

The intervention protocols among the 64 analyzed studies were categorized into single-session and multiple-session designs. Eighteen studies employed a single-session intervention design, while the remaining 46 studies utilized repeated intervention protocols, with treatment durations ranging from 10 days to 12 weeks. Analysis of the intervention duration distribution revealed that “4 weeks” (32 studies) was the most common treatment period, followed by “2 weeks” (5 studies). Furthermore, the durations of “3 weeks,” “10 days,” and “12 weeks” were each adopted by only one study. Notably, none of the 64 studies included a follow-up period. Among the 64 studies, 51 employed conventional manual acupuncture (MA), 10 used electro-acupuncture (EA), and 3 combined MA with EA.

### 3.5 Clinical outcome measures

**Figure 3:**
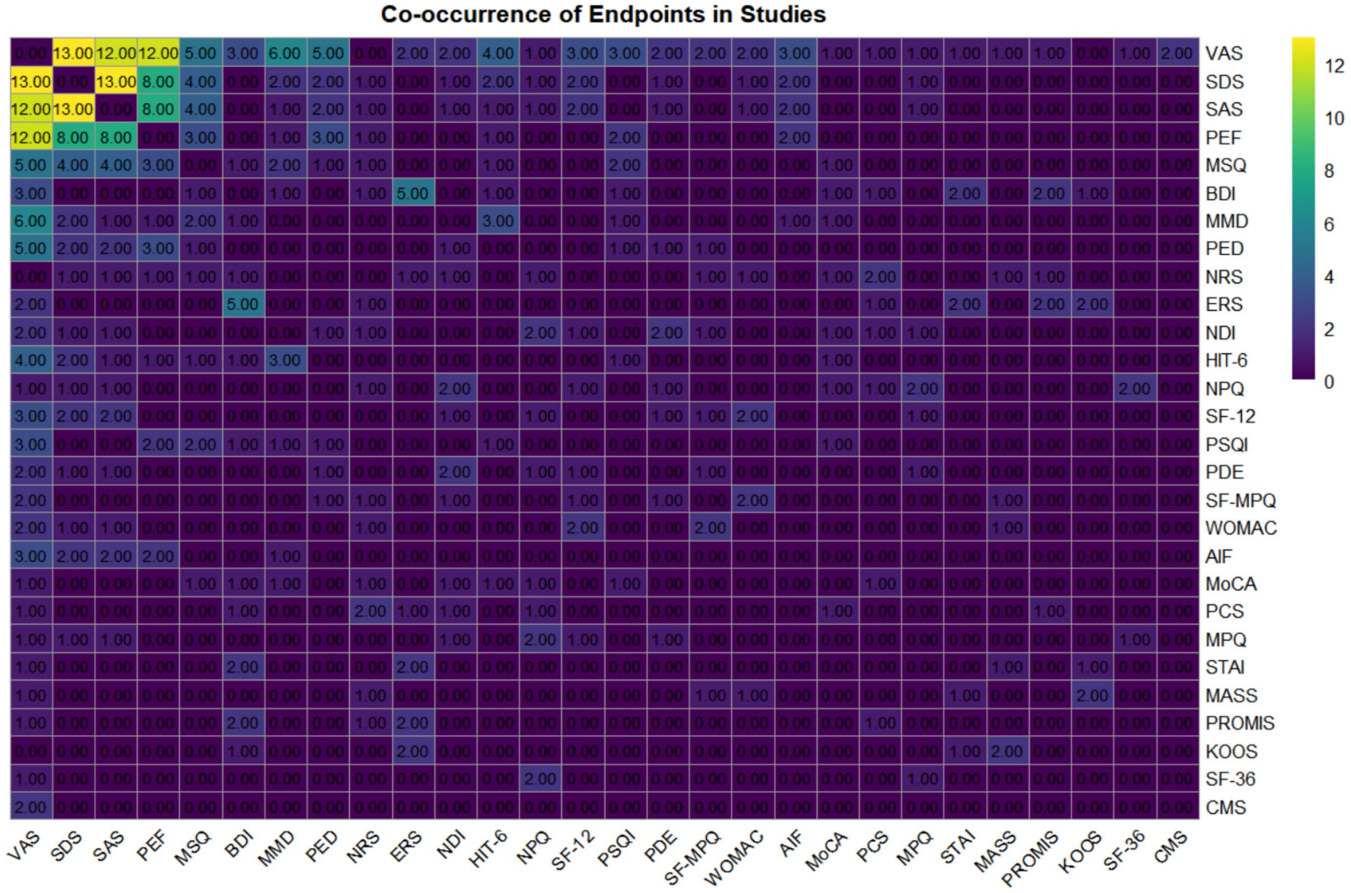
Heatmap matrix of study endpoint co-occurrence (counts: 0–13).

All 64 included studies used fMRI as the core neuroimaging assessment tool. Of these, 8 studies employed fMRI alone, while the remaining 56 additionally included a total of 64 other clinical outcome measures. Statistical analysis of indicator combinations reported more than once (Figure 3) revealed that the combination of “VAS and SDS” and “SDS and SAS” were the most frequent (13 occurrences each), followed by “VAS and SAS” and “VAS and PEF” (12 occurrences each).

### 3.6 Neuroimaging analysis metrics

Regarding brain imaging evaluation strategies, 50 studies adopted a single analytical perspective (48 used rs-fMRI, one study using structural MRI and one using t-fMRI) Fourteen studies employed multimodal integration strategies, among which 5 combined rs-fMRI and structural MRI, and 9 combined rs-fMRI and t-fMRI.

The 64 studies involved 14 distinct neuroimaging data processing methods. In terms of frequency of use, functional connectivity (FC, 35 studies), regional homogeneity (ReHo, 14 studies), and independent component analysis (ICA, 10 studies) were the most prevalent. General linear model (GLM, 9 studies), amplitude of low-frequency fluctuation (ALFF, 6 studies), fractional ALFF (fALFF, 5 studies), degree centrality (DC, 4 studies), and voxel-based morphometry (VBM, 4 studies) were also commonly applied. Methods including diffusion tensor imaging (DTI), dynamic FC (dFC), dynamic functional network connectivity (dFNC), voxel-mirrored homotopic connectivity (VMHC), dynamic ALFF (dALFF), and Granger causality analysis (GCA) were each used in no more than 2 studies.

### 3.7 Brain region alterations

**Figure 4:**
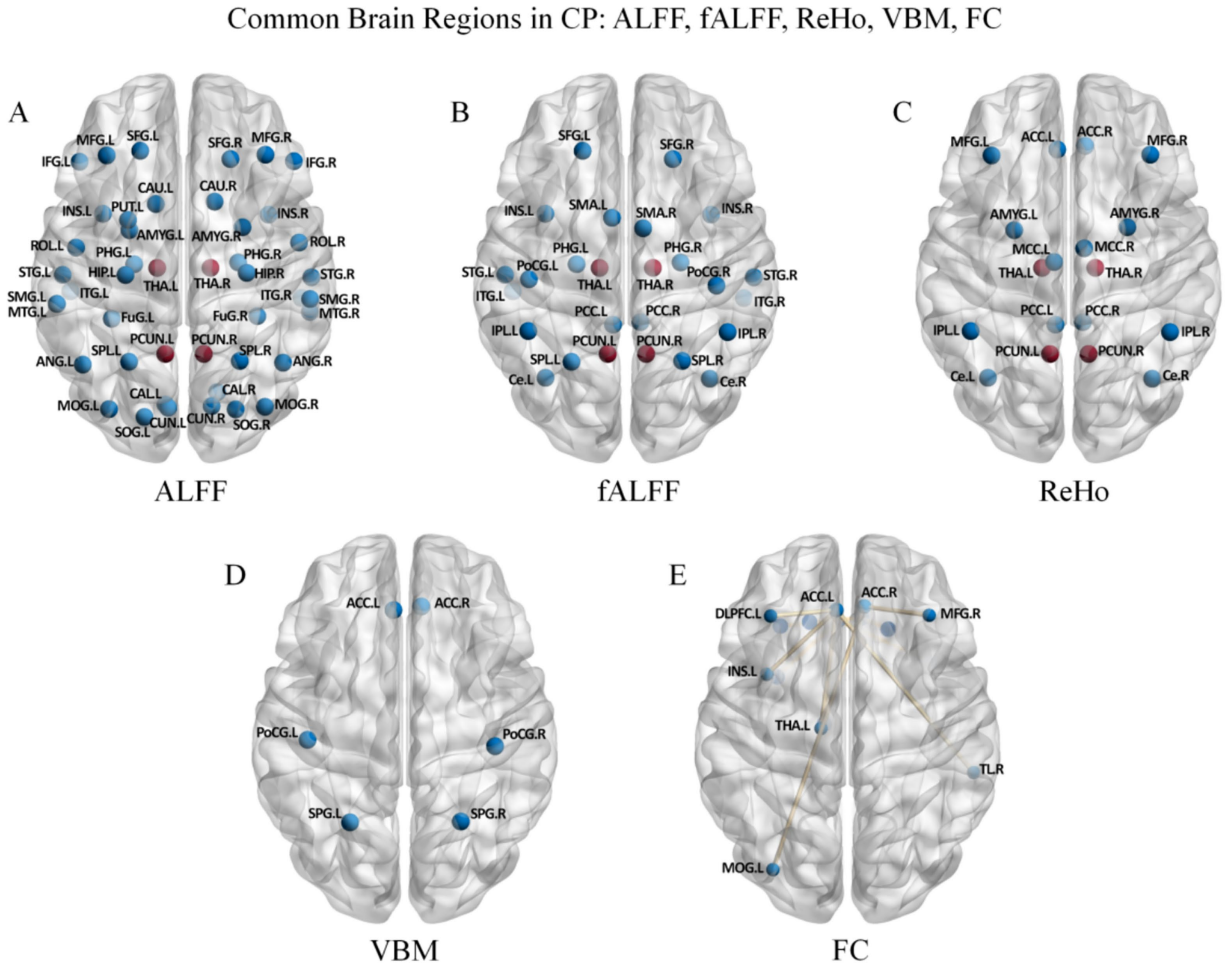
ALFF, fALFF, ReHo, VBM, and FC common/differential brain regions in CP vs. HC. Panels represent findings based on different neuroimaging metrics: **(A)** ALFF, **(B)** fALFF, **(C)** ReHo, **(D)** VBM, and **(E)** FC. Red-highlighted regions in panels **A–C** indicate brain regions commonly identified across ALFF, fALFF, and ReHo analyses.

**Figure 5:**
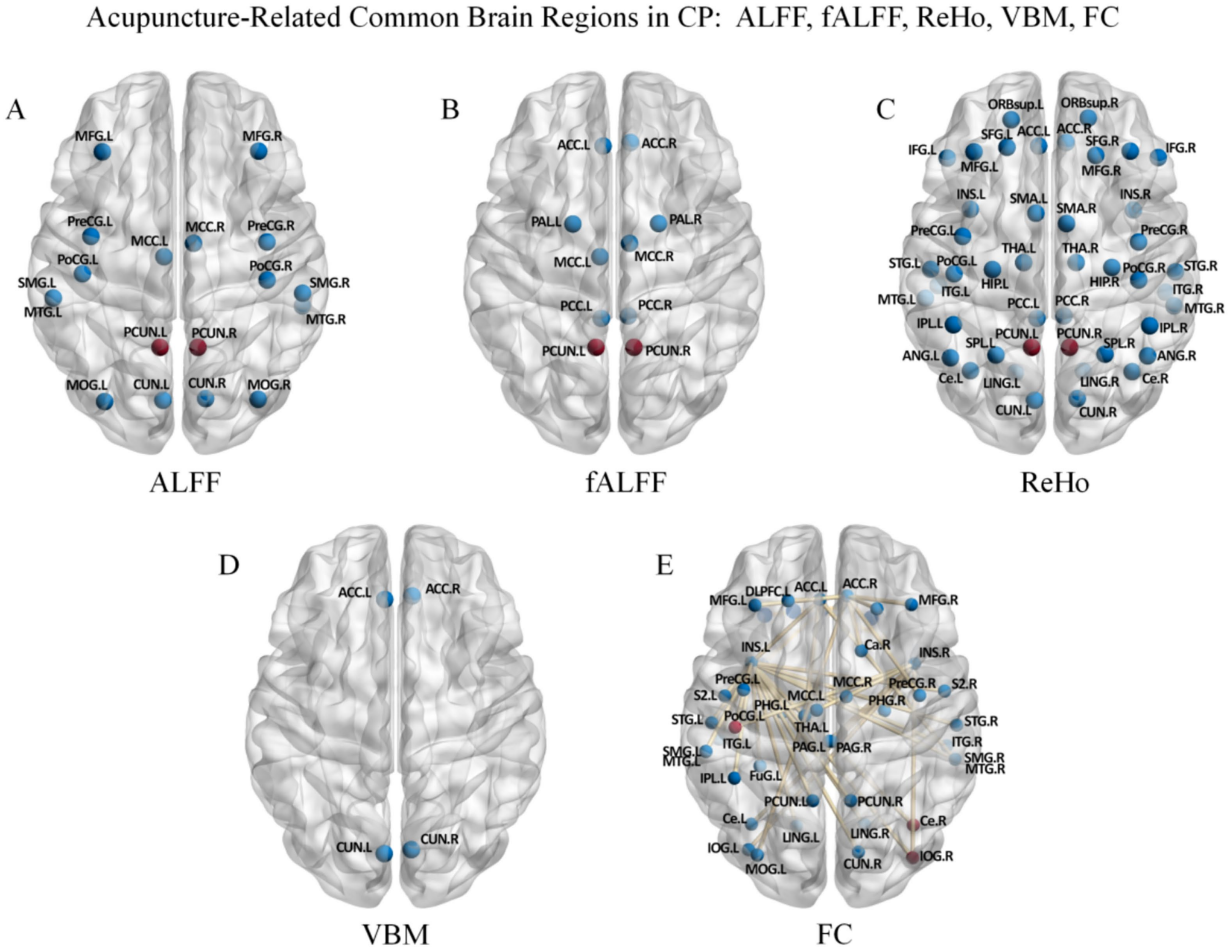
ALFF, fALFF, ReHo, VBM, and FC common/differential brain regions in CP before vs. after acupuncture intervention. Panels represent findings based on different neuroimaging metrics: **(A)** ALFF, **(B)** fALFF, **(C)** ReHo, **(D)** VBM, and **(E)** FC. Red-highlighted regions in panels **A–C** indicate brain regions commonly identified across ALFF, fALFF, and ReHo analyses. Red-highlighted regions in panel E indicate brain regions reported in two or more FC findings.

**Figure 6:**
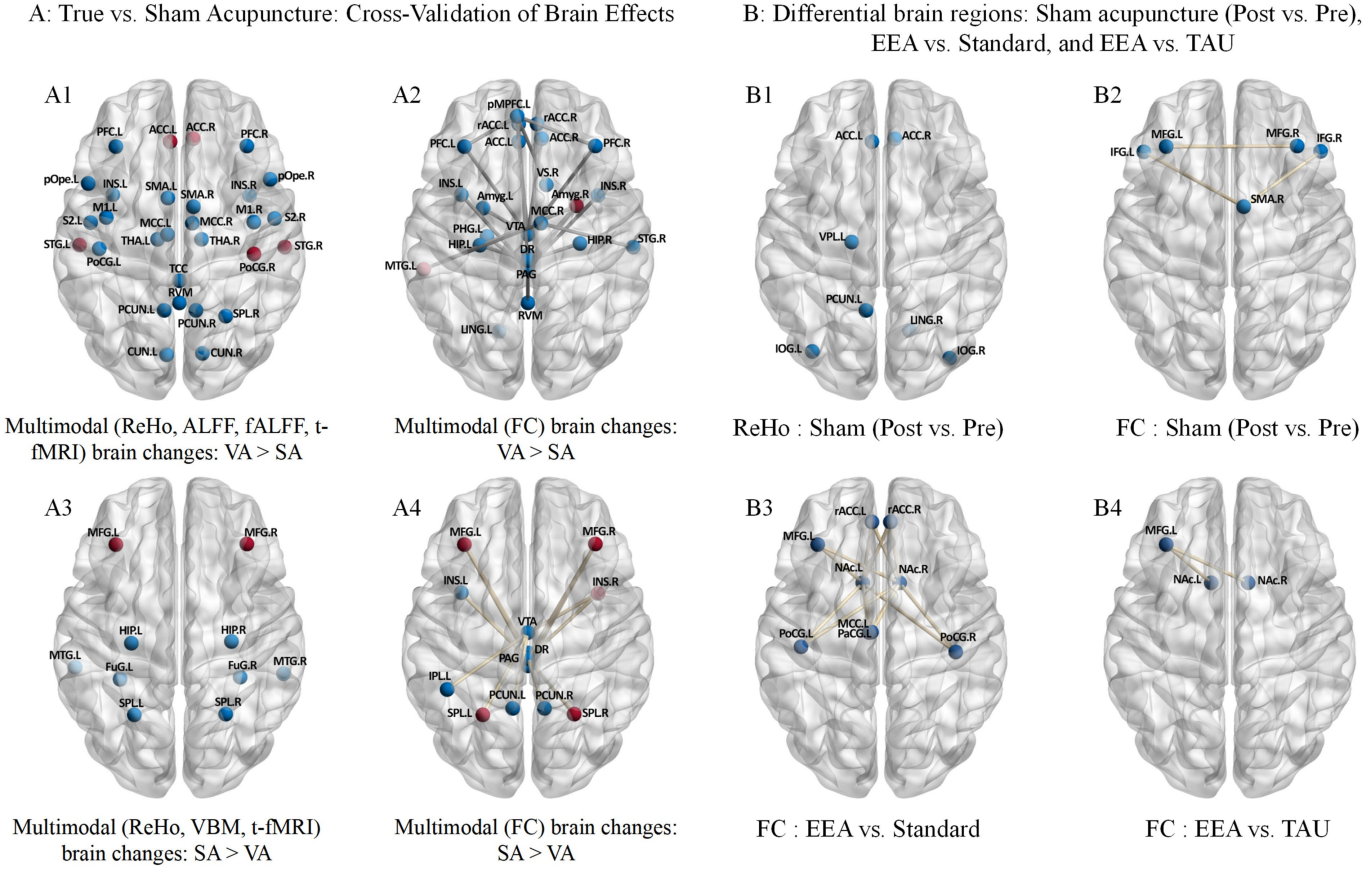
Multimodal common/differential brain regions across acupuncture-related comparisons in CP. **(A1)** Multimodal brain changes (ReHo, ALFF, fALFF, and t-fMRI): VA > SA. **(A2)** Brain changes based on FC: VA > SA. **(A3)** Multimodal brain changes (ReHo, VBM, and t-fMRI): SA > VA. **(A4)** Brain changes based on FC: SA > VA. **(B1)** ReHo changes: sham acupuncture (post vs. pre). **(B2)** FC changes: sham acupuncture (post vs. pre). **(B3)** FC changes: EEA vs. standard acupuncture. **(B4)** FC changes: EEA vs. TAU. Red-highlighted regions indicate differential brain regions supported by two or more contributing findings within the corresponding panel, including repeated findings across imaging metrics in multimodal panels or repeated findings within the same analytical category, as applicable.

To systematically clarify the central response patterns to acupuncture intervention in patients with CP, this study integrated regional brain differences reported in the existing literature using various sMRI and rs-fMRI analysis methods, and summarized significant results from five of the most common and representative approaches: FC, ReHo, ALFF, fALFF, and VBM. The integration encompassed three aspects: (1) disease baseline characteristics, i.e., regional brain differences between CP patients and HC (Table 2 and Figure 4); (2) overall longitudinal effects of acupuncture intervention, which integrated results from observational studies and RCTs that focused solely on pre- versus post-intervention changes (Table 3 and Figure 5); and (3) specific differential central responses based on rigorously controlled designs, for which 13 RCTs were included, with particular emphasis on longitudinal changes following sham acupuncture, comparisons and cross-validation designs between verum and sham acupuncture, as well as central changes induced by different intervention modalities such as EEA and conventional treatment (Table 4 and Figure 6). Given the considerable heterogeneity in seed region selection for FC analyses, this study, in the discussion of the first and second parts, only included core seed regions reported with high frequency in the literature for focused analysis, in order to ensure the robustness and representativeness of the conclusions. Meanwhile, other less frequently applied methods were excluded, such as DC, DTI, dFC, and GCA. Notably, in the subsequent summary figures, co-occurring target regions cross-validated by two or more imaging metrics or analytical methods are uniformly highlighted in red to emphasize their robustness and specificity as core central response nodes.

**Table 2:** Brain regions with significant differences: chronic pain vs. HC (by anatomical structure).

Analysis method	Core node	Significant aberrant brain regions
FC	ACC.L	INS (INS.L) FL (DLPFC.L) TL (TL.R) Dien (THA.L)
	ACC.R	FL (MFG) OL (MOG.L)
ReHo	–	INS (INS) FL (SFG, SMA) PL (PCun, SPL, IPL, PoCG) TL (STG, ITG, PHG) LL (PCC) Dien (THA) Ce (Ce)
ALFF	–	INS (INS) FL (SFG, MFG, IFG, ROL) PL (PCun, SPL, SMG, ANG) OL (CUN, SOG, MOG, Pericalcarine Cortex) TL (STG, MTG, ITG, FuG, PHG, HIP) Gray Matter Nuclei (Put. L, Ca, Amyg) Dien (THA) BS (RVM, TCC)
fALFF	–	FL (MFG) PL (PCun, ANG) LL (ACC, MCC, PCC) Gray Matter Nuclei (Amyg) Dien (THA) Brainstem (RVM) Ce (Ce)
VBM	–	PL (SPG, PoCG) LL (ACC)

**Table 3:** Brain regions with significant differences: pre- vs. post-acupuncture in chronic pain (by anatomical structure).

Analysis method	Core node	Significant aberrant brain regions
FC	ACC.L	INS (INS.L) FL (DLPFC.L) TL (TL.R) Dien (THA.L)
	ACC.R	FL (MFG, SFG.R, PreCG.R) OL (MOG.L) Gray Matter Nuclei (Ca.R)
	INS.L	FL (PreCG.L) PL (PCun, PoCG.L, SMG.L, IPL.L, S2) TL (STG, MTG, ITG, FuG.L) OL (LING, IOG.R, CUN.R) Ce (Ce.R)
	INS.R	PL (PoCG.L) TL (PHG) OL (IOG) LL (MCC) BS (PAG) Ce (Ce.L)
ReHo	–	INS (INS) FL (SFG, MFG, IFG, PreCG, ORBsup, SMA) PL (PCun, SPL, IPL, ANG, PoCG) OL (CUN, LING) TL (STG, MTG, ITG, HIP) LL (ACC, PCC) Dien (VPM, VL, THA) BS (Pons) Ce (Ce)
ALFF	–	FL (MFG, PreCG) PL (PCun, SMG, PoCG) OL (CUN, MOG) TL (MTG) LL (MCC) Brainstem (bilateral RVM, TCC)
fALFF	–	PL (PCun, SPL, PoCG) LL (ACC, MCC, PCC) Gray Matter Nuclei (PAL)
VBM	–	OL (CUN) LL (ACC)

**Table 4:** Brain regions with significant differences: specific responses in controlled trials (by anatomical structure).

Grp./Con.	Analysis method	Core node	Significant aberrant brain regions
VA > SA	FC	DR	OL (LING.L) Dien (THA.R)
		MR	Gray Matter Nuclei (Amyg.R) INS (INS) TL (PHG.L)
		VTA	LL (ACC) FL (PFC) Gray Matter Nuclei (Amyg.L)
		PAG	Gray Matter Nuclei (Amyg.R) BS (RVM) TL (HIP)
		Amyg.R	TL (MTG.L)
		MCC.R	TL (MTG.L, STG.R)
		pMPFC.L	BS (PAG) LL (rACC) FL (PFC) Gray Matter Nuclei (VS.R)
	ReHo	–	FL (SMA) TL (STG) LL (ACC) Dien (THA)
	ALFF	–	BS (RVM, TCC)
	fALFF	–	PL (SPL.R, PoCG.R)
	t-fMRI	–	INS (INS) FL (M1, PFC) PL (PoCG, pOpe, S2, PCun) OL (CUN) TL (STG) LL (ACC, MCC)
SA > VA	FC	DR	FL (MFG.L)
		MR	FL (MFG.R)
		VTA	PL (SPL.L, IPL.L, PCun.L) INS (INS.R)
		PAG	PL (PCun.R, SPL.R) INS (INS) FL (MFG)
	ReHo	–	FL (MFG) TL (HIP, MTG)
	VBM	–	FL (MFG.L)
	t-fMRI	–	FL (MFG) PL (SPL) TL (FuG)
Sham (Post vs. Pre)	FC	MFG. R	FL (MFG.L)
		SMA. R	FL (IFG)
	ReHo	–	PL (PCun.L) OL (LING.R, IOG) LL (ACC) Dien (VPL.L)
EEA > Standard	FC	NAc	LL (MCC.L, PaCG.L, rACC) PL (PoCG) FL (PFC.L)
EEA > TAU	FC	NAc	FL (MFG.L)

#### 3.7.1 Baseline central abnormalities in chronic pain

As shown in Table 2, in baseline comparisons between CP patients and HC, ReHo, ALFF, and fALFF analyses consistently revealed significant abnormalities in the THA and PCun of patients (highlighted in red in Figures 4A–C) as shared regions across ALFF, fALFF, and ReHo analyses.

#### 3.7.2 Overall central modulatory effects of acupuncture intervention

With respect to longitudinal changes following acupuncture treatment (Table 3 and Figure 5), multiple regional activity measures, including ALFF, fALFF, and ReHo, demonstrated significant pre- versus post-intervention differences in the PCun (highlighted in red in Figures 5A–C). Furthermore, FC analyses repeatedly identified the PoCG. L, Ce. R, and IOG. R as target regions exhibiting significantly altered functional connectivity (highlighted in red in Figure 5B).

#### 3.7.3 Specific neuroimaging features of acupuncture treatment

The RCTs employing rigorously controlled designs revealed intervention-specific responses (Table 4). In cross-validation analyses where verum acupuncture elicited significantly greater changes than sham acupuncture (VA > SA), the MTG. L and Amyg. R showed significant FC alterations across multiple studies (highlighted in red in Figure 6A2); additionally, the ACC and STG were jointly implicated by ReHo and t-fMRI, while the PoCG. R received convergent support from fALFF and t-fMRI (highlighted in red in Figure 6A1). Conversely, in studies where SA > VA, the SPL, MFG and INS. R exhibited significant changes in multiple FC analyses (highlighted in red in Figure 6A4); notably, alterations in the MFG were corroborated by three distinct modalities—ReHo, VBM, and t-fMRI—underscoring its pronounced structural and functional sensitivity to sham acupuncture (highlighted in red in Figure 6A3).

## 4 Discussion

This scoping review systematically synthesized 64 fMRI studies on acupuncture for CP, covering disorders such as MIG, KOA, and CLBP. By integrating participant characteristics, acupuncture intervention parameters, neuroimaging methodologies, and clinical outcomes, the review provides a preliminary overview of CP-related central abnormalities, acupuncture-associated brain responses, and the methodological heterogeneity of the current literature. In interpreting the present findings, cross-sectional patient-versus-healthy-control comparisons were considered primarily informative of CP-related baseline abnormalities, pre-post comparisons of acupuncture-associated changes, and randomized controlled comparisons of differential central responses under distinct intervention conditions.

Methodological heterogeneity remains a major challenge. According to Desmond et al., fMRI studies generally require at least 12 participants per group, and this criterion has also been supported by previous international reviews of acupuncture neuroimaging research (Desmond and Glover, 2002; Beissner, 2011). However, sample sizes varied substantially across the included studies, and some did not meet this recommended standard, which may reduce comparability across studies. In addition, because the central neural mechanisms of CP are closely related to disease duration, inconsistent duration criteria further increase sample heterogeneity and limit interpretability and reproducibility (Apkarian et al., 2005). At the study design level, most included studies focused on immediate analgesic responses or short-term cumulative effects, and none incorporated post-intervention follow-up, making it difficult to assess the persistence and stability of acupuncture-related brain changes. Heterogeneity may also arise from intervention parameters, since treatment duration and stimulation mode, such as manual acupuncture versus electroacupuncture, may influence the intensity, extent, and pattern of central responses (Yan et al., 2020; Lin et al., 2020; Li et al., 2006; Kong et al., 2002). Notably, the distribution of outcome measures suggests that the combined assessment of pain intensity and mental health status has become an important trend in current research.

Current multimodal fMRI evidence indicates that, relative to HC, patients with CP exhibit widespread network-distributed functional abnormalities, along with potential structural alterations in several brain regions, which are mainly concentrated within the DMN and SMN (Zeng et al., 2025). By analyzing the methodological specificity of different neuroimaging metrics, we can more systematically elucidate the multidimensional impairment mechanisms of the DMN and SMN. Multiple core nodes of the DMN present significant abnormalities (Figure 4). The PCun shows alterations not only in regional spontaneous neural activity and its synchrony (ReHo, ALFF) but also in interregional FC. The coexistence of local functional dysregulation and impaired global information integration suggests a possible association with deficits in self-awareness and episodic memory construction observed in patients with CP (Osborne et al., 2021). Aberrant ReHo in the PCC may also disrupt self-perception and emotional integration processes (Murillo et al., 2023). Notably, baseline ALFF characteristics of the PCC have been reported to correlate with the prediction of acupuncture analgesic efficacy, indicating that the intrinsic functional state of DMN core nodes may be an important factor influencing therapeutic sensitivity (Gao et al., 2025a). Furthermore, the ACC exhibits not only state-dependent abnormalities in FC but also pathological structural alterations as assessed by VBM. The coexistence of transient functional fluctuations and gray matter morphological abnormalities suggests that the interactive imbalance between the emotional pain processing network and cognitive control network may extend beyond short-term functional dysregulation, although the presence of durable structural–functional remodeling remains to be established (Wang X. et al., 2025; Wang Y. et al., 2025; Yu et al., 2025). Additionally, combined abnormalities in FC and ALFF within the MFG reflect state-dependent functional adjustments of neural substrates related to emotional regulation in CP patients, and may reflect repeated or treatment-related functional adjustments (An et al., 2025).

The SMN also exhibits significant abnormalities in CP, with pathological changes characterized by the coexistence of functional state imbalance and structural alterations in several brain regions (Figure 4). Specifically, the PoCG shows concurrent abnormalities in both ReHo and VBM, while the SPG also presents altered VBM. This overlap of functional dysregulation and structural morphological change suggests that CP not only impairs sensorimotor integration but may also be accompanied by structural–functional alterations of potential longer-term relevance in core somatosensory brain regions (Chaikla et al., 2025; Anderson et al., 2025). Furthermore, concurrent abnormalities in ReHo and DC in the SMA indicate disrupted local neuronal synchrony and reduced global network hub efficiency, respectively, which may impair motor preparation and the function of descending pain modulatory pathways (Gombaut and Holmes, 2022; Jenkins et al., 2000; Kong et al., 2024). As important components of the higher-order somatosensory association cortex (Figure 4D), disrupted ReHo in the SPL and IPL indicates dysregulation of somatosensory attention allocation and spatial information processing under pain conditions (Wei et al., 2024; Garin et al., 2022). Combined abnormalities in FC and ReHo in the Ce further suggest impaired local processing stability and interregional coordination in sensory prediction and motor coordination (Nguyen and Person, 2025). Collectively, multimodal imaging findings indicate that patients with CP exhibit widespread functional deficits in the somatosensory and motor central processing systems, accompanied by relatively stable structural changes in several brain regions (Wu et al., 2009).

Furthermore, core brain regions directly involved in the pain matrix and limbic system exhibit coordinated pathological changes across multiple neuroimaging dimensions (Figure 4). Within the INS, abnormalities in ALFF and ReHo indicate disrupted local spontaneous activity and synchrony, while altered FC with multiple brain regions reflects impaired interregional communication. This dysregulation across multiple metrics suggests that CP may simultaneously impair the sensory integration function of the posterior insula and the emotional regulation function of the anterior insula (Molnar-Szakacs and Uddin, 2022; Uddin et al., 2017). Similarly, the THA not only exhibits widespread local functional abnormalities (ALFF, fALFF, and ReHo) but also shows altered FC with the ACC. Previous studies have indicated that the THA-ACC axis jointly participates in pain–emotion integration processing (You et al., 2022), and may further constitute a broader emotion regulation network with brain regions such as the amygdala and hippocampus, thereby reflecting the aberrant central transformation of nociceptive signals into emotional distress (Wang X. et al., 2025; Wang Y. et al., 2025; Fontaine et al., 2025; Henn et al., 2023). Moreover, abnormal local amplitude metrics (ALFF/fALFF) in regulatory nodes of the limbic system and brainstem (e.g., the RVM) provide indirect support for potential state-dependent functional decline in the descending inhibitory pathway (Wang X. et al., 2025; Wang Y. et al., 2025; Costa et al., 2024; Allen et al., 2021; Tan and Kuner, 2021).

Collectively, multimodal imaging evidence suggests that the central pathophysiology of CP involves widespread functional abnormalities within and across large-scale brain networks rather than isolated regional dysfunction. Local metrics such as ALFF/fALFF and ReHo primarily reflect state-dependent alterations in regional spontaneous activity and local neural synchrony (Zang et al., 2004; Lee et al., 2013), which may be related to deficits in sensory discrimination and emotional processing, whereas FC reflects interregional coupling (Biswal et al., 1995) and indicates that these local abnormalities may be embedded within broader large-scale network imbalance. At the intra-network level, multiple studies have reported reduced activity and connectivity within the DMN in patients with CP (Li et al., 2014; Zou et al., 2019a; Li et al., 2012). However, such indices primarily reflect functional states at specific time points and are insufficient on their own to support inferences about permanent structural change. In addition, how abnormalities in different intra-network pathways differentially relate to pain perception, memory, and emotion remains to be clarified. At the level of network interaction, most current studies focus on FC among a limited number of functional systems, such as the ECN, SN, DMN, and SMN (Li et al., 2017a; Li et al., 2015). Both the DMN and SMN play important roles in CP (Hidalgo-Lopez et al., 2025), but how they jointly shape the multidimensional clinical manifestations of pain through dynamic functional integration remains insufficiently understood. Further investigation of these interaction mechanisms, particularly the relationship between short-term functional abnormalities and possible longer-term network and structural alterations, may help clarify the complex neuropathological basis of CP.

Analysis of pre- versus post-acupuncture fMRI changes showed that the brain regions modulated by acupuncture overlapped substantially with regions identified as abnormal in CP, mainly within the DMN and SMN, while also involving key nodes of the limbic system and pain matrix. This correspondence suggests that acupuncture-related brain changes may occur, at least in part, in regions implicated in CP-related dysfunction, particularly those involved in pain perception, sensory processing, and emotional regulation, and may reflect state-dependent functional modulation rather than fixed directional effects (Kim et al., 2020; Tian et al., 2021; Li et al., 2020; Hashmi et al., 2014; Zhou et al., 2023). However, it remains unclear whether the multidimensional improvement associated with acupuncture is mediated by coordinated changes across multiple active regions or is driven predominantly by a smaller number of key nodes. Studies included in this review further suggest that brain regions with predictive value for acupuncture efficacy are mainly concentrated in the DMN and its connected regions (Tu et al., 2019; Yang et al., 2020; Li et al., 2020). This observation supports the view that the DMN may represent a candidate network of interest for future response-prediction studies (Xu J. et al., 2022), while also suggesting that interactions within and between functional brain networks may constitute an important pathway through which acupuncture modulates CP.

**Figure 7:**
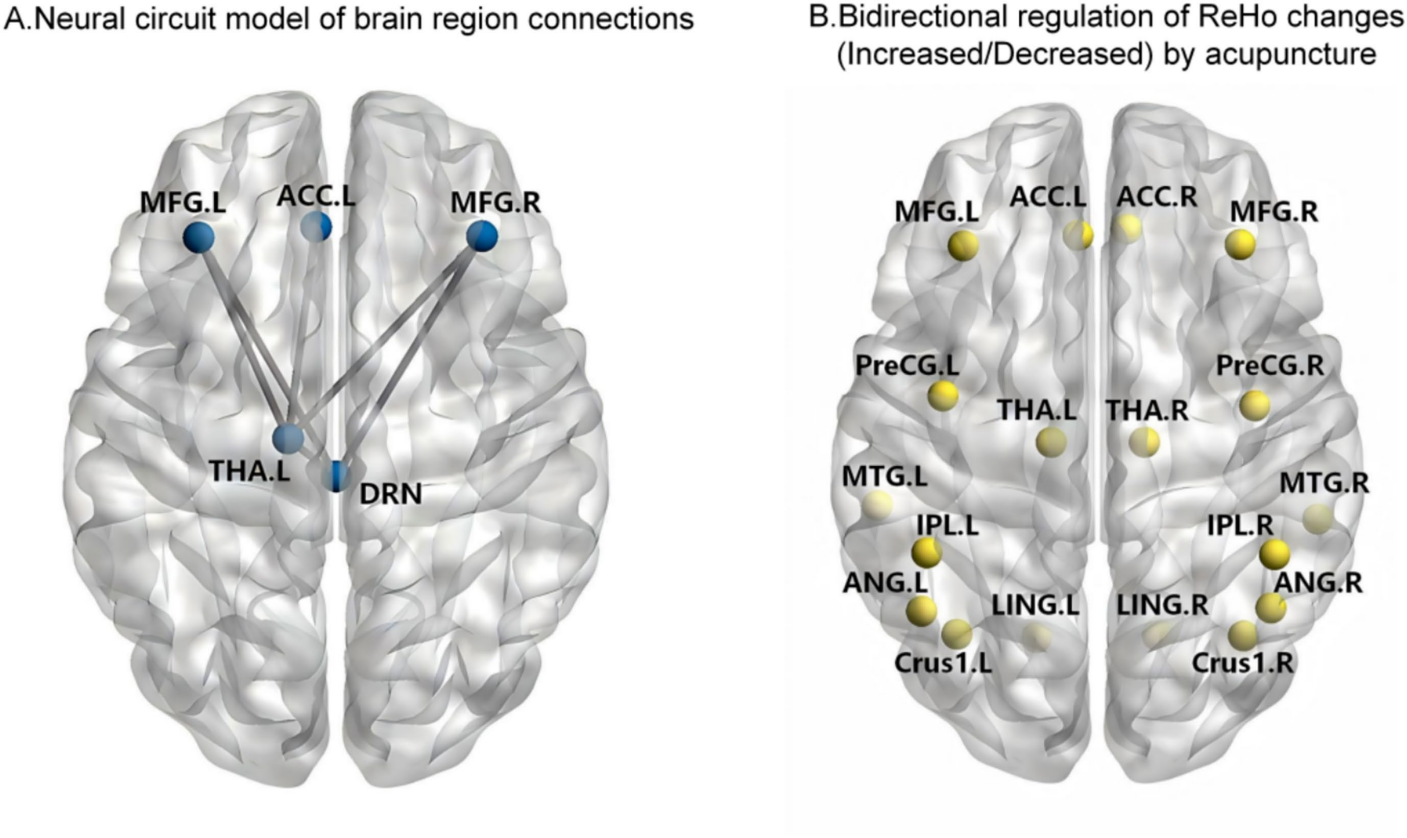
Acupuncture-induced neural circuit and bidirectional ReHo changes in chronic pain. **(A)** Neural circuit model of brain region connections. **(B)** Bidirectional regulation of ReHo changes, including increased and decreased ReHo changes, by acupuncture.

Based on previous multimodal evidence, this study proposes an exploratory multi-node neural circuit hypothesis model, whose topological pathways are as follows (Figure 7A): ACC.L – THA.L – DRN – MFG – THA.L – ACC.L (Shen et al., 2021a; Wang et al., 2024; Hou et al., 2025). Each node in this hypothesis model plays a specific role in pain and emotional regulation: the ACC is a key node in maintaining pain hypersensitivity and regulating emotion (Lee et al., 2022; Liu et al., 2025); the THA is a crucial hub for somatosensory transmission and affective cognition (You et al., 2022), and its abnormal connectivity constitutes an important pathological basis of CP. Acupuncture may be associated with a shift of thalamocortical network organization toward a less dysregulated pattern, potentially through modulation of time-varying functional connectivity between the THA and the DMN and SMN (Hou et al., 2025; Quan et al., 2024; Tu et al., 2020; Isenburg et al., 2021); the DRN, as a core serotonergic regulatory center (Zhang et al., 2024a; Zhang Y. N. et al., 2024), may exhibit abnormal activation in the CP state, and EA has been reported to be associated with reduced pain-related anxiety alongside changes in functional integrity within the rACC-DRN pathway (Wu et al., 2024; Zhou et al., 2019; Lee M. J. et al., 2019); the MFG, acting as a higher-order hub integrating pain perception and emotion, may exhibit impaired ability to coordinate multidimensional pain information due to abnormal functional connectivity (Mosch et al., 2023; Li et al., 2025). Taken together, these findings support a preliminary hypothesis that acupuncture-related functional connectivity changes may involve an ACC-centered circuit of potential relevance to pain and emotion processing (Tu et al., 2019; Egorova et al., 2015).

In ReHo analyses, acupuncture intervention was associated with changes in local neural synchrony across multiple brain regions (Figure 7B), including the THA, PrG, ACC, Ce, brainstem, pons, LING, AG, IPL, MFG, and MTG. Notably, the direction of ReHo changes in these brain regions shows high heterogeneity across studies—the same brain region may exhibit either increased or decreased local synchrony in different literatures (Chen W. C. et al., 2015; Hou et al., 2014; Zhang et al., 2018; Jia et al., 2021a; Jia R. H. et al., 2021; Qin et al., 2019; Zhang Y. N. et al., 2024; Liu et al., 2021; Li et al., 2023; Zhao et al., 2014). This bidirectional regulatory feature may not merely reflect inter-study inconsistency; instead, it may partially represent the state dependency of acupuncture’s central effects (Sack et al., 2024). Meanwhile, such directional differences could be jointly influenced by multiple factors, including disease status, stimulation protocols, scanning timing, and data processing strategies (Zhang et al., 2021a; Huang et al., 2012). Accordingly, we propose a working hypothesis entitled “state-dependent neurodynamic homeostasis”. Acupuncture-related modulation of regional ReHo may predominantly manifest as being associated with a shift of aberrant brain functional states toward relatively more balanced patterns, rather than reflecting a fixed directional response (Egorova et al., 2015). Existing evidence suggests that acupuncture-induced brain effects are not fixed unidirectional responses, but are more likely to represent a neuromodulatory process that evolves dynamically throughout intervention (Sack et al., 2024; Bai et al., 2009); their effect size and direction may be partially shaped by pre-treatment baseline brain states, together with evolving changes in brain network function during treatment (Tu et al., 2019; Yang et al., 2020). Conceptually, this hypothesis corresponds to the traditional Chinese medicine principle of “reinforcing deficiency and reducing excess,” which highlights bidirectional homeostatic modulation (Zhang et al., 2021b). Within this framework, baseline ReHo values may represent a preliminary imaging feature of interest for future response-prediction studies. Future prospective stratified studies combining baseline ReHo, rsFC, and other multimodal imaging markers are warranted to clarify whether the directional changes of post-acupuncture brain activity correlate with pretreatment brain states, and whether the degree of functional normalization is associated with clinical benefits (Yu et al., 2020).

At the network level, these regions are primarily distributed across the SMN, DMN, and CCN (Wu et al., 2009; Raichle and Snyder, 2007; Shen et al., 2015; Jiao et al., 2020), suggesting that acupuncture may exert its therapeutic effects by dynamically coordinating the functional states of these networks. Notably, in the present multimodal metric analysis, the PCun is the only brain region that exhibits significant alterations across multiple resting-state fMRI dimensions at baseline and following acupuncture intervention, encompassing local metrics such as ALFF, fALFF, and ReHo, as well as FC (Cai et al., 2020; Ning et al., 2020; Zhao et al., 2014; Qu et al., 2021; Zou et al., 2019b). As a core node of the DMN, the PCun may represent a region in which acupuncture-related changes are observed in patients with CP (Osborne et al., 2021), and the degree of restored intra-network connectivity is correlated with pain duration (Fiúza-Fernandes et al., 2025). Moreover, acupuncture-related brain responses may involve dynamic changes spanning regions from the RVM to the central amygdala and the PCun, with possible spatiotemporal accumulation characteristics (Chen et al., 2022). Meanwhile, abnormalities in local activity metrics of the PCun coexist with disrupted intra- and inter-network FC across the whole brain, suggesting its potential involvement in the impairment of global functional integration capacity of the brain (Fiúza-Fernandes et al., 2025). The ACC represents another critical brain region exhibiting significant alterations in VBM and interregional FC at baseline and after acupuncture treatment (Shen et al., 2021a; Yang et al., 2020; Li et al., 2023; Zhao et al., 2014; Xu et al., 2021a). As a key region for multisensory integration and emotional processing, structural and functional abnormalities of the ACC are strongly associated with various CP conditions, and patients with CP present marked coupling abnormalities between gray matter morphology and FC (Ou et al., 2024; Zhang et al., 2023). Collectively, these findings suggest that acupuncture may preferentially engage brain regions showing marked abnormalities in CP, and that changes in local neural activity, neuronal synchrony, and gray matter morphology may be accompanied by alterations in interregional FC and large-scale brain network organization, although durable structural–functional remodeling remains to be confirmed.

A key interpretive consideration in acupuncture neuroimaging research is that sham acupuncture should not be regarded as a physiologically inert control (Lim and Go, 2024), because depending on stimulation modality and anatomical location, it may still activate cutaneous and muscular afferents, engage segmental or descending pain modulatory pathways, and recruit expectancy-related cortical processes (Fan et al., 2023; Crawford et al., 2023). In the multimodal evidence for SA > VA (Figures 6A3,A4), the MFG exhibited relatively prominent multimodal alterations of potential adaptive relevance, manifested as task-based activation during superficial stimulation at non-acupoints, as well as significant changes in resting-state ReHo, FC, and gray matter structure (Wang et al., 2024; Makary et al., 2018; Zhao et al., 2014; Chen et al., 2014). Further within-group longitudinal analysis (pre- vs. post-sham acupuncture) revealed significant changes in FC between bilateral MFG (Figure 6B2) (Gao et al., 2025b), that the response of the MFG to sham acupuncture may not be limited to transient activation and short-term alterations, but may also involve changes related to cognitive control across the intervention period, potentially accompanied by a tendency toward broader network-level adjustment (Tu et al., 2021; Habermann et al., 2025). Given that the MFG is a key node of the FPN (Marti et al., 2025), its significant connectivity alterations with crucial brainstem hubs intimately involved in endogenous descending pain modulation (including the DR, MR, and PAG) (Wang et al., 2024; Egorova et al., 2015; Zhang et al., 2024b; Liang and Labrakakis, 2024) indicate that sham acupuncture-induced brain responses are not confined to local sensory processing, but may also involve cross-level coupling between higher-order cognitive control systems and brainstem pain modulatory systems. Concurrently, FC between cortical regions such as the INS, PCun, and SPL and midbrain hubs including the VTA and PAG also exhibited significant differences (Yu et al., 2020). From a functional specialization perspective, the INS is implicated in interoception and salience encoding (Craig, 2009), the SPL participates in tactile spatial localization and somatosensory integration (Huang et al., 2022), and the PCun is associated with contextual monitoring and self-referential processing (Dadario and Sughrue, 2023), whereas the VTA and PAG are, respectively, involved in reward anticipation processing and descending analgesic modulation (Zhang et al., 2024b; Zhang Y. N. et al., 2024; de Jong et al., 2022). The aforementioned connectivity patterns suggest that sham acupuncture-induced brain responses may result from the combined influence of superficial peripheral sensory input and higher-order central regulation (Crawford et al., 2023). This further implies that superficial stimulation, non-penetrating stimulation, or non-acupoint stimulation, although lacking the deep tissue stimulation and characteristic *deqi* afference specific to verum acupuncture, should not be equated with physiologically inert stimuli (Lim and Go, 2024); rather, such stimulation may still generate peripheral sensory input capable of further central nervous system processing through the activation of cutaneous mechanoreceptors and afferent fibers associated with superficial muscle and fascia (Fan et al., 2023).

In contrast, in the multimodal evidence for VA > SA (Figures 6A1,A2), verum acupuncture tended to be associated with a relatively more focused pattern of central modulation associated with stronger deep afference and further involving pain sensory encoding, emotional–motivational evaluation, and descending analgesic systems. Notably, the ACC exhibited relatively prominent multimodal alterations of potential adaptive relevance, with significantly enhanced FC with the VTA and left pMPFC, as well as significant differences in ReHo and task-based activation during acupuncture (Yu et al., 2020; Makary et al., 2018; Zhao et al., 2014). These findings suggest that, compared with sham acupuncture, verum acupuncture may not only induce transient nociceptive/salience responses but may also be associated with modulation of prefrontal–limbic networks related to pain appraisal, cognitive control, and reward–motivation processing across the intervention period (Yu et al., 2020; Song et al., 2024). Concurrently, the PoCG exhibited significant enhancement in both task-based activation and fALFF (Gollub et al., 2018; Wei et al., 2024), indicating that verum acupuncture may be more likely to engage the somatosensory discrimination system relative to sham acupuncture (Ziegler et al., 2023). This finding aligns directionally with the characteristics of verum acupuncture, which involves skin penetration, deep tissue stimulation, and more typical *deqi*-associated afference (Choi et al., 2025), suggesting that its brain effects may not be restricted to the non-specific processing elicited by superficial stimulation but may more fully reflect the central encoding of peripheral sensory input (Fan et al., 2023). Meanwhile, the STG and MTG play important roles in emotion-related multisensory integration (Choo et al., 2025), and the enhanced FC of STG–MCC.R, MTG.L–Amyg.R, and MTG.L–MCC.R (Liu L. et al., 2022) suggests that verum acupuncture may strengthen the integration between sensory information and the emotional–motivational system. More importantly, FC between the Amyg and the MR, VTA, and PAG was significantly enhanced (Wang et al., 2024; Yu et al., 2020), suggesting that verum acupuncture may more strongly engage limbic–brainstem pain modulatory pathways centered on the amygdala relative to sham acupuncture. The amygdala and VTA are key nodes for pain emotion–motivation and reward–motivation, respectively (Xu et al., 2026; Fitzgerald and Day, 2025); the PAG is a critical hub of the classical descending pain modulatory system (De Preter and Heinricher, 2024); and the MR, as part of the raphe nuclei, has its serotonergic system implicated in pain and aversion/emotion processing (Kawai et al., 2022).

Further evidence from EEA also suggests a potential contribution of contextual–expectancy components to acupuncture-related brain responses (Figures 6B3,B4). In the available comparisons, relative to standard acupuncture or usual care, EEA was mainly associated with altered functional connectivity centered on the NAc, involving cingulate/prefrontal regions and some somatosensory-related areas (Kong et al., 2018). Given that the NAc is a key hub for reward anticipation, motivational salience, and expectancy-related valuation (Baliki et al., 2010), this pattern is more suggestive of an amplification of reward–motivational and contextual appraisal components embedded in treatment, rather than a mere repetition of sensory input effects. Collectively, these findings suggest that VA, SA, and EEA may engage partly overlapping but not identical neural processes: SA may more strongly reflect superficial sensory input with partial cognitive–expectancy engagement, EEA may be more strongly associated with reward- and context-related processing, whereas the relative advantage of VA may lie in the additional recruitment of more directed somatosensory and limbic–brainstem pain modulatory pathways. At present, however, the relationship between acupuncture-related imaging changes and clinical improvement remains insufficiently resolved, and the observed brain responses should not be interpreted as direct causal drivers of symptom relief.

## 5 Limitations

Although this scoping review was conducted using a systematic approach to literature identification and synthesis, several limitations should be acknowledged. First, substantial heterogeneity existed across the included studies in terms of study design, participant characteristics, acupuncture protocols, control settings, imaging processing methods, and sample sizes. Moreover, several critical details essential for study replicability and interpretation of findings were generally insufficiently reported, and the methodological quality of the included studies varied considerably. These factors may have reduced the comparability and scientific interpretability of the findings. Second, the available evidence was derived predominantly from Asian populations and was frequently based on relatively small sample sizes, thereby limiting the external validity of the conclusions and their generalizability to different ethnicities and more diverse populations. Third, evidence capable of substantiating long-term neuroplastic remodeling remains insufficient. None of the 64 included studies incorporated a long-term follow-up design, and only a limited number of studies employed structural neuroimaging metrics such as VBM and DTI. Consequently, the observed alterations in FC, ReHo, ALFF, and fALFF can only be cautiously interpreted as acupuncture-related or state-dependent functional fluctuations, rather than definitive evidence of sustained structural and functional brain reorganization. Finally, the control protocols varied substantially across studies, and sham acupuncture does not constitute an entirely physiologically inert stimulus, making it difficult to precisely isolate the specific central effects attributable to verum acupuncture. In addition, machine learning-based studies on efficacy prediction remain at a preliminary stage, and the candidate imaging biomarkers identified to date still lack sufficient cross-validation and external validation. Future studies should address these limitations through better-powered multicenter designs, clearer control stratification, standardized acupuncture reporting, and multimodal longitudinal neuroimaging approaches.

## 6 Conclusion

The evidence synthesized in this scoping review indicates that the central pathophysiological features of CP are primarily characterized by functional imbalances at the brain network level, involving aberrant interactions among the DMN, SMN, and pain- and emotion-related brain regions including the ACC, PCun, INS, and THA. Existing multimodal fMRI findings further suggest that acupuncture intervention may be associated with modulation of key nodes linked to CP-related abnormalities, together with functional changes across multiple levels encompassing local brain activity, local neural synchrony, and interregional FC. Concurrently, comparative evidence from controlled studies indicates that the central processes engaged by verum acupuncture, sham acupuncture, and enhanced-expectancy acupuncture may partially overlap yet remain distinct: sham acupuncture-related responses are more likely to involve superficial sensory input and partial cognitive–expectancy components; enhanced-expectancy acupuncture engages to a greater extent reward and contextual appraisal processing represented by NAc-related connectivity; whereas verum acupuncture may show a relative tendency toward additional engagement of pathways associated with somatosensory discrimination, pain appraisal, emotional–motivational regulation, and brainstem descending analgesic systems. Taken together with the bidirectional ReHo changes and the recurrently observed imaging alterations in ACC- and PCun-related circuits, this review proposes a cautious working framework: the central effects of acupuncture may predominantly manifest as a state-dependent recalibration process of brain networks, namely, being associated with adjustment of dysregulated brain states toward relatively more balanced functional configurations. It is important to emphasize that this framework should be regarded as a hypothetical interpretation derived from available neuroimaging evidence, rather than definitive proof of causal mechanisms or enduring neuroplastic remodeling. Given the substantial heterogeneity among existing studies, the absence of long-term follow-up, and the fact that current evidence is derived primarily from Asian populations, extrapolation of acupuncture-related central mechanisms to broader and more diverse populations warrants considerable caution. Future large-sample, multicenter, longitudinal, and multimodal studies—particularly those conducted across different regions and populations—are warranted to test and refine this framework, and to further elucidate the relationship between acupuncture-related brain responses and clinical improvement in CP.

## Data Availability

The original contributions presented in the study are included in the article/Supplementary material, further inquiries can be directed to the corresponding author.
