# Supplementary material for: Functional brain alterations associated with acupuncture for chronic pain: a scoping review of fMRI studies
Source: Front Neurosci. 2026 May 21;20:1819418. doi: 10.3389/fnins.2026.1819418 (PMC13233432; doi:10.3389/fnins.2026.1819418)
Supplement: Supplementary file 1 [file Data_Sheet_1.PDF]

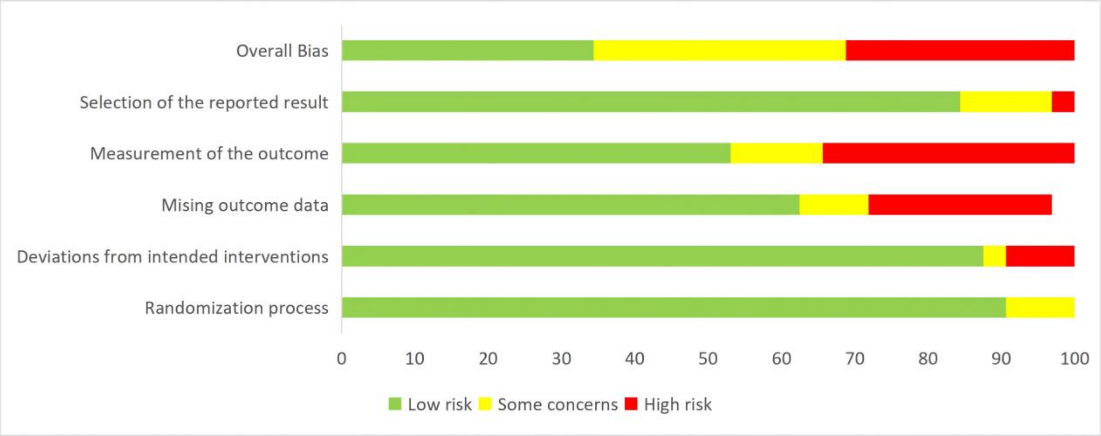

Supplementary Figure S1. RoB 2 summary of included RCTs.

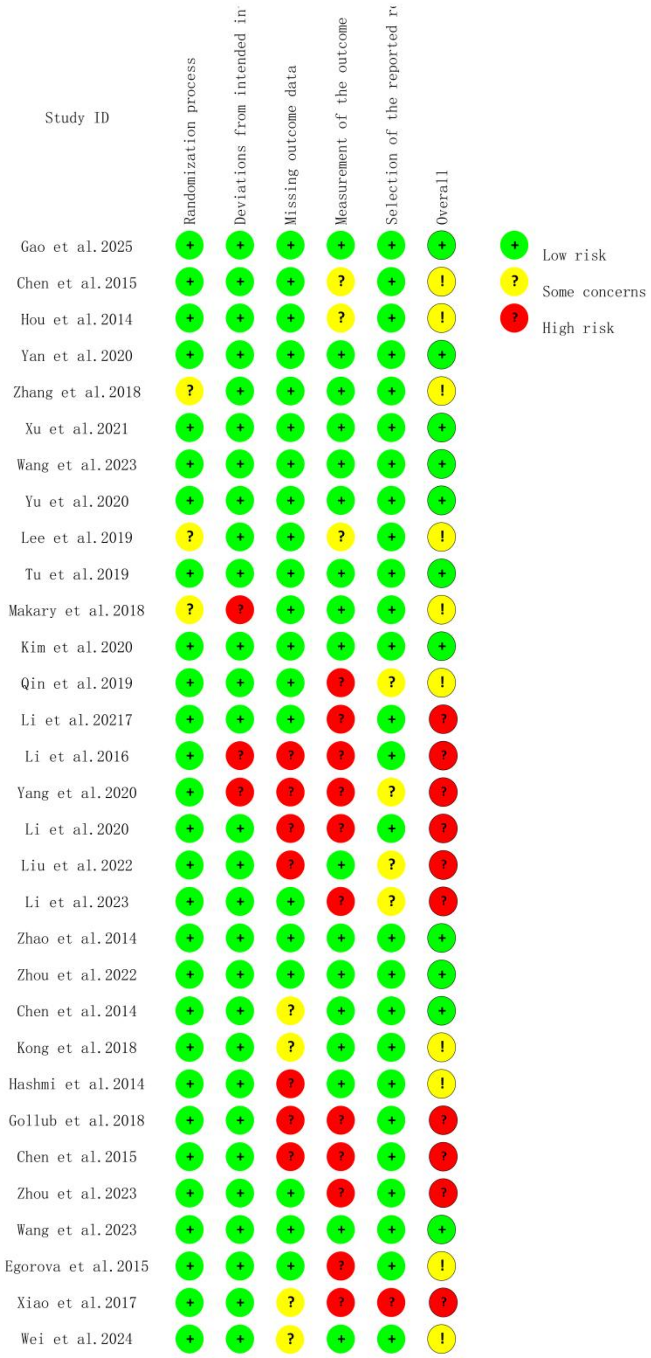

Supplementary Figure S2. RoB 2 traffic-light plot of included RCTs.

Supplementary Table 1 Reporting compliance to the STRICTA statement

Criteria	Item	Description	Total （n=64）	%	Chinese （n=28）	%	English （n=36）	%
Acupuncture rationale	1a	Style of acupuncture	64	100	28	100	36	100
	1b	Reasoning for treatment provided	56	87.5	24	85.71	32	88.89
	1c	Extent to which treatment was varied	63	100	28	100	35	97.22
Needling details	2a	Number of needle insertions per participant per session	59	92.18	24	85.71	35	97.22
	2b	Names of points used (or location if nonchannel points) unilateral or bilateral	62	96.88	28	100	34	94.44
	2c	Depth of insertion	43	67.19	20	71.42	23	63.89
	2d	Response sought (e.g., de qi)	51	79.69	20	71.42	31	86.11
	2e	Needle stimulation	64	100	28	100	36	100
	2f	Needle retention time	54	84.38	25	89.28	29	80.56
	2g	Needle type	39	60.94	18	64.28	21	58.33
Treatment regimen	3a	Number of treatment sessions	64	100	28	100	36	100
	3b	Frequency and duration of treatment sessions	64	100	28	100	36	100
Other components of treatment	4a	Details of other interventions administered to the acupuncture group	2	3.13	1	3.57	1	2.86
	4b	Setting and context of treatment	60	93.75	28	100	32	88.89
Practitioner background	5	Description of participating acupuncturists	38	59.38	16	57.14	22	61.11
Control interventions	6a	Rationale for the control or comparator in the context of the research question	21	32.81	2	7.14	19	52.78
	6b	Precise description of the control or comparator	35	54.69	13	46.42	22	61.11

Supplementary Table 2 Methodological Quality Assessment of Included Acupuncture-Related fMRI Studies Using the Newcastle-Ottawa Scale (NOS)

Reference	Study design	Selection	Comparability	Exposure/Outcome	Total
Effect of acupuncture on pain-emotion related brain regions in patients with cervical spondylosis of cervical type: a fMRI study	Case-control study	**		*	***
Effects of acupuncture at Fengchi(GB20)on resting-state functional MRI in patients with cervical spondylosis of artery type	Single-group pretest-posttest study	*		*	**
Study on Analgesic Effect of Acupuncture on Cervical Spondylosis by MRI of Resting State	Case-control study	**		*	***
Population screening for acupuncture treatment of neck pain:a machine learning study	Case-control study	***		*	****
Modulation effect of acupuncture treatment on chronic neck and shoulder pain in female patients: Evidence from periaqueductal gray-based functional connectivity	Case-control study	***	**	*	******
Objective evaluation on brain network imaging of "treating same disease with different methods" effect of acupuncture for chronic low back pain (cLBP)	Case-control study	***	**	*	******
Impact of Acupuncture on Brain Functional Connectivity Network in Patients with Low Back Pain	Single-group pretest-posttest study	*		*	**
Acupuncture treatment of chronic low back pain reverses an abnormal brain default mode network in correlation with clinical pain relief	Case-control study	***	**	*	******
Effects of Electroacupuncture on Shuaigu on Pain-related Functional	Case-control study	***	**	*	******

Reference	Study design	Selection	Comparability	Exposure/Outcome	Total
Network of Migraine Patients:a Task-fMRI Study					
Clinical Efficacy and Regulation of Brain Region of Differentiation of Meridians Acupuncture Based on the Theory of"Gen-Jie"in Migraine Patients without Aura	Single-group pretest-posttest study	*		*	**
Effect of acupuncture on default mode network in patients with migraine based on functional Magnetic Resonance Imaging: a preliminary study	Single-group pretest-posttest study	*		*	**
Prediction of Therapeutic Effect of Acupuncture on Migraine Without Aura Based on Dynamic Functional Connection of Thalamus	Single-group pretest-posttest study	**	**	*	*****
Research on brain function network of patients with migraine without aura by acupuncture based on graph theory	Case-control study	***	**	***	********
Effects of acupuncture at Zulinqi(GB41) on pain related brain networks of migraine patients: An f MRI study	Case-control study	***	**	**	*******
Effect of acupuncture on migraine without aura: a resting-state functional magnetic resonance study	Case-control study	***	**	***	********
Study on the Influence of Acupuncture Zulinqi(GB41)on the Amplitude of Low Frequency Oscillation of Migraine	Case-control study	***	**	**	*******
Acupuncture at Zulinqi (GB41) on Brain Degree Centrality in Patients with Migraine Without Aura: An fMRI Study	Case-control study	***	**	**	*******
Study of resting-state functional MRI on immediate effect regulated by	Single-group pretest-posttest	*	**	***	******

Reference	Study design	Selection	Comparability	Exposure/Outcome	Total
acupuncture on acupoints in patients with migraine without aura	study				
Abnormal thalamocortical network dynamics in patients with migraine and its relationship with electroacupuncture treatment response	Case-control study	***	**	***	********
Acupuncture Modulation Effect on Pain Processing Patterns in Patients With Migraine Without Aura	Prospective Cohort	***	**	***	********
Acupuncture reversible effects on altered default mode network of chronic migraine accompanied with clinical symptom relief	Case-control study	***	**	***	********
Acupuncture treatment modulates the resting-state functional connectivity of brain regions in migraine patients without aura	Prospective Cohort	***	**	***	********
The cumulative therapeutic effect of acupuncture in patients with migraine without aura: Evidence from dynamic alterations of intrinsic brain activity and effective connectivity	Prospective Cohort	***	**	***	********
Differential Modulating Effect of Acupuncture in Patients With Migraine Without Aura: A Resting Functional Magnetic Resonance	Prospective Cohort	***	**	***	********
The effects of acupuncture treatment on the right frontoparietal network in migraine without aura patients	Prospective Cohort	***	**	***	********
Resting-state brain function connectivity in patients with knee osteoarthritis treated with acupuncture based on continuous edge analysis	Prospective Cohort	***	**	***	********
Clinical efficacy evaluation and central mechanism study of acupuncture in treating chronic knee osteoarthritis	Prospective Cohort	***	**	***	********

Reference	Study design	Selection	Comparability	Exposure/Outcome	Total
Acupuncture Enhances Dorsal Raphe Functional Connectivity in Knee Osteoarthritis With Chronic Pain	Prospective Cohort	***	**	***	********
Decreased intrinsic brain connectivity is associated with reduced clinical pain in fibromyalgia	Prospective Cohort	***	**	***	********
Central mechanism of analgesic effect of Huaisanzhen on L5 nerve root pain based on functional magnetic resonance imaging	Prospective Cohort	***	**	***	********
Effects of Acupuncture on Default Mode Network Images of Chronic Sciatica Patients in the Resting Network State	Prospective Cohort	***	**	***	********
Influence of Acupuncture Analgesia on Cerebral Function Imaging in Sciatica Patients	Single-group pretest-posttest study	*	*	**	****
Changes in resting-state functional connectivity in nonacute sciatica with acupuncture modulation: A preliminary study	Prospective Cohort	***	**	***	********
